# Case of Excision of a Scirrhous Parotid Gland

**Published:** 1816-06

**Authors:** 


JOURNAL AND REVIEW.

VOL. I.] JUNE, 1816. [NO. 6.

### PART I.

### ORIGINAL COMMUNICATIONS.

### *Case of Excision of a scirrhous Parotid Gland.*

(Communicated by Dr. PALMER.)

THE parotid gland occupies the deep recess between the ramus of the lower jaw and the auditory and mastoid processes of the temporal bone. In form, it is somewhat triangular; in outline, ill-defined. Its facial border adheres to the internal pterygoid muscle; its auricular dips behind the anterior margin of the sterno-cleido-mastoid. Its inferior extremity descends below the angle of the jaw. It is covered externally by the cervical fascia, platysma myoides, and skin. The styloid process of the temporal bone forms its internal boundary. While deeply buried in the centre of the gland, the external carotid artery sends off important branches to the jaws, the face, the temple, and the ear. The portio dura of the seventh pair, or great facial nerve, traverses its substance in passing from the stylo-mastoid orifice of the cranium to the cheek. Almost in contiguity with its internal surface are, the internal carotid artery, jugular vein, and the nerves accompanying them; the eighth, accessory, glosso-pharyngeal, and great sympathetic.

Any operation for the removal of an organ thus situated and connected, few surgeons will contemplate without alarm, or enter upon without anxiety and trepidation. Such indeed are the dangers and difficulties which beset it, that several of the ablest surgeons of our own time have reprobated the attempt as desperate, or denounced it as utterly impracticable. Of these, it may suffice to enumerate the names of Abernethy,* Bell,† and Allan Burns:‡ and most of my readers will probably concur in regarding as more than commonly perilous the operation upon which that man, to whom exclusively belongs the glory of having first tied the carotid and external iliac arteries, would turn his back. But some exaggerated sense of danger; some hypothetical phantom, the aerial nature of which it is my object to demonstrate, seems to have haunted the imagination of these gentlemen, and crossed and disturbed their intellectual vision, while contemplating this subject. I do not, at the present moment, recollect any work wherein excision of the parotid gland is *minutely* recorded or described.

Two principal arguments have been adduced by those who reprobate this operation, in support of their opinions. First, the intimate connexions of the external carotid artery with the gland. To such an objection, at a period memorable, like the present, in the annals of British surgery, reply is almost superfluous. What surgeon will now dread the consequences of destroying the external carotid artery, or tremble at hæmorrhage from any vessel accessible to the scalpel and ligature? The second objection is more specious: no one, it is contended, can clear away the substance of the parotid from all the deep recesses to which it penetrates; and imperfect removal of the organ, when invaded by malignant disease, must be useless, as the progress of the poison and reproduction of the morbid

---

* "No one surely would think of cutting out the parotid gland."—*Mr. Abernethy.*

† "The cutting out completely the parotid gland is a thing *quite impossible*, since the greatest of all the arteries, viz. the temporal and the maxillary, lie absolutely imbedded in the gland."—*Bell's Anatomy*, vol. ii. page 293. Yet, inexplicably enough, Mr. John Bell boasts of having *frequently performed* this operation.

‡ "Extirpation (of the parotid gland) is quite out of the question. Its impracticability is proved by reviewing the connexions of the gland."—*Burns' Surgical Anatomy*, page 267. Again, "I have endeavoured to place the question regarding the extirpation of the parotid gland in its proper light, and to shew, from the anatomy of its connexions, that it is an operation which, *in no situation, and under no circumstances, ought to be undertaken.*" Same Work, page 270. And yet Mr. Burns talked, with perfect coolness, of *tearing out an enlarged thymus!*

action will not thereby be cut off or obviated. From the former part of this proposition, none who are correctly acquainted with the anatomy of the angle of the jaw will dissent. That the doctrine broached in the latter is unsound, as the practice to which it leads destructive, I am prepared to prove. One solitary fact, in elucidation of this point, is worth a host of arguments. By all who feel interested in the solution of an important surgical problem, the following narrative of successful excision of a scirrhous parotid gland will, perhaps, be favourably received.

In the early part of the year 1811, I was requested by a stout, athletic, middle aged man, to examine a swelling, which, some months before had arisen, without evident cause, below the left ear. It occupied precisely the site, and apparently implicated the structure, of the parotid gland. It possessed the volume of a pigeon's egg; was immoveable; exceedingly hard and irregular on its surface. The integuments covering it were loose and natural, except over one of the central eminences, where adhesion and a slightly puckered appearance of the skin were exhibited. A sensation of numbness was constantly felt in the seat of the disease, and occasionally "stinging pains." The numbness sometimes extended to the corresponding cheek. The progress of the tumor, at first slow, had recently been much more rapid. No derangement in the structure or functions of the internal organs was perceptible. Under these circumstances, I hesitated not to propose an operation, and to urge its immediate performance, ere the neighbouring glands should become contaminated by the evidently malignant affection of the parotid, or the constitution suffer from extension of its ravages. But this advice, before given in vain by another surgeon, was at once rejected; and I saw nothing more of the man for some months.

About the commencement of 1812, I was requested, in passing the house, to visit him again. The tumor was now so much increased in bulk as seriously to impede the motions of the lower jaw. The numbness and lancinating pains were greatly aggravated; the knotty irregularities of the surface more distinctly marked; the integuments of the centre rapidly verging towards ulceration. Indigestion and slight febrile paroxysms indicated the approaching ravages of the local malady on the system. The man's physiognomy had assumed a deep expression of disquietude and alarm. The dreadful fate, which want of courage and resolution must very soon entail upon him, was depicted in strongest colours; the affecting claims of a wife and helpless family, urged with all the earnestness and energy which such an occasion was calculated to inspire. At length, he declared himself ready to submit to any measures that we might propose; and Sunday, the 19th of January, was fixed for the operation.

On that day, the knife was taken by the gentleman whose opinion had first been solicited by the patient; and with whom I had previously a long conversation respecting the case. The morbid portion of integument was, in the first place, insulated by two curved incisions meeting each other, above and below, at an acute angle. Upon exposing the surface of the organ towards the masseter muscle, all hope that the enlargement implicated exclusively the lymphatic gland imbedded in its centre,* at once vanished. The substance of the parotid itself, indubitably recognized by the origin of its excretory duct, was seen knotty and enlarged. Our minds had been duly prepared for this discouraging circumstance: our resolution was irrevocably fixed. The integuments, covering the posterior part of the gland, were now dissected back. On detaching it towards the mastoid process, the posterior artery of the ear was divided; and bled, for a time, profusely. The temporal, transverse facial, and internal maxillary, successively sprang on insulating the gland in other directions. Tbe hæmorrhage was great; but pressure on the mouths of the vessels prevented it from becoming excessive. At length, the whole of the morbid organ was detached, except at the base. This was the precise period for the application of a ligature; but the hope of speedily terminating a bloody and protracted operation prevailed with us over every other consideration. The final detachment of the gland was effected by a stroke of the scalpel, and the gush from the external carotid was most appalling. I thrust a sponge into the wound, and retained it with considerable force. The man, whose countenance had long been bleaching, now became faint. The operator, availing himself of this favourable moment, fixed a tenaculum into the artery. I attempted to secure it, but this was rendered exceedingly difficult by the depth and narrowness of the wound: the ligature slipped. Two other attempts were

---

* See the review of Mr. Allan Burns' "Surgical Anatomy," page 61 of our present volume. Mr. Burns asserted that, in most cases of supposed parotic enlargement, the disease will be found to implicate exclusively the substance of this little central gland.

equally abortive. The man was now recovering from his syncope; the artery again beginning to pour out a frightful torrent. The situation of the poor fellow was, at this moment, most critical; our own embarrassing. At last, amid such a deluge of blood as I hope that these hands may never again be imbued in, the ligature was fortunately fixed. No return of hæmorrhage was provoked by the exhibition of a cordial, and repeated ablution with warm water. After careful examination of the tumor, to ascertain that the whole of the morbid substance had been removed, the edges of the wound were drawn together, and retained by plaster and bandage: and, after a few hours' repose, the man was sufficiently recovered to bear the fatigue of conveyance to his own home, distant two miles.

The mass removed was as large as a hen's egg, and weighed upwards of two ounces. Internally, it displayed the fibrous and radiated structure characterizing real scirrhus. Nothing remarkable occurred during the cicatrization of the wound, which was accomplished in a few weeks.

On visiting our patient shortly after the operation, I was surprised to see the right angle of the mouth drawn, and his visage distorted, as in hemiplegia. He complained also, that the left side of the face was destitute of sensibility. A moment's reflection threw light upon the source of this phænomenon. The trunk of the left facial nerve having been cut out with the parotid gland, the supply of nervous energy to the corresponding facial muscles was necessarily interrupted: hence they could no longer exert an antagonist power. But the insensibility and distortion gradually wore off;* and scarcely a trace of the deformity once existing now remains.

Successfully as this operation terminated, I would by no means recommend that it should again be performed in a similar manner. Much of the loss of blood—much of the embarrassment and peril, incurred in this instance, might be avoided by the simple mode of procedure which I shall cursorily describe:—After fairly exposing the parotid gland with the scalpel, that instrument should, *as much as possible*, be laid aside, and the tumor detached from its connections by working with the fingers and knife-handle. Thus insulated to its base, a strong ligature should be passed tightly around it, and secured. The gland may

---

* Probably as the supply of nervous influence became re-established by other channels.

then be left to slough away like a strangulated polypus; or the operation may at once be terminated by dividing its root with the scalpel, *externally* to the ligature, in the mode proposed by the late Sir Charles Blick, for the removal of diseased axillary glands. The sufferings of the patient would probably be much mitigated, if the trunk of the facial nerve, directly at its exit from the skull, were cut through in an early stage of the operation. This, to the surgeon correctly acquainted with the anatomy of the parts, would prove no difficult undertaking;* and to all, except well-informed and expert operators, the region around the angle of the jaw is, or should be, forbidden ground.

Four years have now elapsed since the operation which constitutes the subject of this narrative, was performed. The man remains well to this day.† Let no one then talk of the impossibility of extirpating the parotid gland! Or, stricken by a sense of imaginary danger, renounce an attempt, which, successful as we have seen it under circumstances of aggravated difficulty, may surely again succeed with greater precaution and more judicious management; and thus doom to a death of accumulated horror the human being whom a little professional energy and courage might have served to rescue.

*Tamworth, March,* 1816.

---

* Rules for dividing the facial nerve are given in a note, page 61 of this volume.

† By the majority of my readers, four years, passed without recurrence of the morbid affection, will be regarded as affording tolerably strong evidence of the success of the operation. That this, however, constitutes no absolute criterion, the following case will demonstrate. In the summer of 1807, I removed a small tumour displaying the characters of scirrhus, from the lower lip of a middle-aged female. The whole morbid substance was cut out; the cure remarkably propitious. She remained well until the autumn of 1814. An attack of ophthalmy, occurring at that period, was subdued by a few doses of submuriate of quicksilver, topical bleeding, and fomentations. Shortly afterwards, she called to consult me respecting the tumor of the glands below the jaw. The features of scirrhus were too strongly marked to be mistaken. The woman is now fast approaching to the term of a miserable existence. Does not this case seem to indicate, contrary to the opinion of Mr. Allan Burns, that there may be *specific irritation* as well as *specific absorption*? Confident I am, that neither central fungus nor ulceration existed in the original scirrhus of the lip.

